# Patient-centered care and geriatric knowledge translation among healthcare providers in Vietnam: translation and validation of the patient-centered care measure

**DOI:** 10.1186/s12913-023-09311-z

**Published:** 2023-04-19

**Authors:** Oluwarantimi Adetunji, David Bishai, Cuong Viet Pham, Janiece Taylor, Ngan Tran Thi, Zainab Khan, Abdulgafoor M. Bachani

**Affiliations:** 1 Johns Hopkins International Injury Research Unit, Health Systems Program, Department of International Health, Johns Hopkins Bloomberg School of Public Health, Baltimore, MD USA; 2 Center for Injury Policy and Prevention Research (CIPPR), Hanoi University of Public Health, Hanoi, Vietnam; 3 Johns Hopkins School of Nursing, Baltimore, MD USA

**Keywords:** Geriatrics, Aging, Nurses, Healthcare quality, Cross-cultural adaptation, Vietnam, Healthcare providers, Knowledge translation, Patient-centered care, Person-centered care

## Abstract

**Background:**

People are living longer, and the majority of aging people reside in low- and middle-income countries (LMICs). However, inappropriate healthcare contributes to health disparities between populations of aging people and leads to care dependency and social isolation. Tools to assess and evaluate the effectiveness of quality improvement interventions for geriatric care in LMICs are limited. The aim of this study was to provide a validated and culturally relevant instrument to assess patient-centered care in Vietnam, where the population of aging people is growing rapidly.

**Methods:**

The Patient-Centered Care (PCC) measure was translated from English to Vietnamese using forward-backward method. The PCC measure grouped activities into sub-domains of holistic, collaborative, and responsive care. A bilingual expert panel rated the cross-cultural relevance and translation equivalence of the instrument. We calculated Content Validity Indexing (CVI) scores at both the item (I-CVI) and scale (S-CVI/Ave) levels to evaluate the relevance of the Vietnamese PCC (VPCC) measure to geriatric care in the Vietnamese context. We piloted the translated instrument VPCC measure with 112 healthcare providers in Hanoi, Vietnam. Multiple logistic regression models were specified to test the a priori null hypothesis that geriatric knowledge is not different among healthcare providers with perception of high implementation compared with low implementation of PCC measures.

**Results:**

On the item level, all 20 questions had excellent validity ratings. The VPCC had excellent content validity (S-CVI/Ave of 0.96) and translation equivalence (TS- CVI/Ave of 0.94). In the pilot study, the highest-rated PCC elements were the holistic provision of information and collaborative care, while the lowest-rated elements were the holistic attendance to patients’ needs and responsive care. Attention to the psychosocial needs of aging people and poor coordination of care within and beyond the health system were the lowest-rated PCC activities. After controlling for healthcare provider characteristics, the odds of the perception of high implementation of collaborative care were increased by 21% for each increase in geriatric knowledge score. We fail to reject the null hypotheses for holistic care, responsive care and PCC.

**Conclusion:**

The VPCC is a validated instrument that may be utilized to systemically evaluate the practice of patient-centered geriatric care in Vietnam.

## Background

People are living longer than previous generations, which reflects the collective advancements in economic development, public health and medicine. [1] Longer lives provide opportunities for individuals to continue productive contributions to their families and communities. However, these opportunities depend on ensuring the optimal health of aging people. [2] The challenge of maintaining functional ability at older ages requires realigning the design of healthcare delivery systems to the social and health needs of aging people, who face increased risk of illnesses and declining functional ability due to their environment and the accumulation of molecular and cellular damage. [3, 4]

Care coordination, an element of patient-centered care, is associated with the reduced risk of socialization of aging adult hospitalization, which refers to the utilization of hospital beds as a substitute for long-term care. [5, 6] By facilitating access to community services and specialized care within and beyond the health system for aging adults, patient-centered care promotes successful transitions into the community, reduces readmissions, and improves patient satisfaction and health outcomes, especially among aging people with multimorbidity. [7, 8]

Failure to provide appropriate care to meet the complex needs of aging people may lead to poor health, care dependency, and social isolation. [1] Consequently, disparities exist in the distribution of good health between populations of aging people, especially in developing countries where most aging people are located. [9] This study aims to provide a validated instrument to assess the provision of patient-centered geriatric care in health facilities across Vietnam, which has a rapidly growing aging population. [10] Vietnam’s national aging survey suggests a gap in the provision of patient-centered geriatric care because 1 in 3 aging adults reported an unmet healthcare need for chronic diseases or sensory impairment, despite receiving clinical treatment for an acute illness or injury in the past year. [11, 12] Vietnamese aging adults who received clinical services in the past year had the same level of unmet need for assistance compared with those who had not sought healthcare in the past year. [11] Similarly, aging adults with multimorbidity had 185% or 224% higher risk of unmet health need after receiving healthcare from public or private healthcare providers, respectively, compared to those with one chronic need. [11]

In Vietnam, recent policies, such as the Decision 2151/QD-BYT of 2015, outline national plans to prioritize patient preferences in healthcare delivery and promote quality improvement interventions to improve patient-centered care. [13] In 2023, Vietnam’s National Assembly approved amendments to the Law on Medical Examination and Treatment that incorporated new provisions for patient-centered care. [14] Although healthcare providers may know about person-centered geriatric care, they may be limited in their capacity to translate geriatric knowledge into the practice of quality care. [15] An assessment of the perception of providers, patients, and caregivers is a widely accepted method to evaluate the delivery of quality care, including patient-centered care. [16, 17] To our knowledge, no study has developed a tool to assess patient-centered care in Vietnamese. This study provides an instrument to assess the practice of patient-centered geriatric care, which is an essential component of quality care that translates geriatric knowledge to action for aging people. [18, 19] While diverse instruments are available for assessing patient-centered care, we selected the Patient-Centered Care (PCC) measure because its development was guided by a comprehensive conceptualization of patient-centered care that was derived from an integrative review of conceptual, empirical, and clinical evidence. [20] The content of the PCC measure covers specific activities that constitute patient-centered care across clinical programs within the context of acute care. [21] We translated, validated the cross-cultural relevance, and piloted the PCC measure among healthcare providers in Hanoi, Vietnam.

## Methods

### The patient-centered care measure

Surveys and rating scales are a type of assessment tools to examine practice processes in a systematic way. [22] The PCC measure is a validated instrument with 20 statements and a response scale that ranges from *not at all* (0) to *very much so (5).* [23] The items describe activities that operationalize patient-centered care in the context of acute care.

The PCC measure grouped activities into three components of patient-centered care, which are holistic, collaborative, and responsive care. Holistic care is reflective of comprehensive care and health promotion for patients. It contains one sub-domain with four items on attending to patients’ needs and a second sub-domain with five items on the provision of information to help patients manage their needs and health conditions. Collaborative care contains seven items that describe activities to facilitate shared decision-making with the patient. The four items for responsive care operationalize the individualization of care within the hospital and after discharge. The PCC measure may be used to assess the fidelity of patient-centered care practice and interventions by healthcare providers. Healthcare providers rate how their daily practices compare with the list of activities that operationalize patient-centered care in clinical settings.

The Content Validity Indexing (CVI) score for the original PCC measure was greater than 0.90 for the three subscales, which indicates that nurse practitioners deemed all the questions to be highly relevant to PCC. [21, 24] Only the KR-20 coefficient values for the collaborative subscale reached the 0.70 criterion for ascertaining the reliability of newly developed measures. [24, 25]

### Cross-cultural translation of the patient-centered care measure

The PCC measure was translated and piloted as part of a larger study, which validated tools to assess the capacity of healthcare providers to provide quality geriatric care in Vietnam. [11] The PCC measure was translated with the forward-backward method, which is a longstanding adaptation method for cross-cultural research. [26, 27] A bilingual native Vietnamese speaker translated the PCC measure from English to Vietnamese. A panel of five bilingual researchers reviewed and revised the translation. A different bilingual translator, who did not see the English version of the PCC measure, translated the revised Vietnamese PCC measure back to English. The English back-translation was compared to the original version of the instrument to detect alterations in meaning.

### Cross-cultural validation of the patient-centered care measure

An expert panel of seven bilingual geriatric experts rated each question of the Vietnamese Patient-Centered Care measure (VPCC) on a scale from 1 to 4 (*not relevant, somewhat relevant, very relevant, or highly relevant*) using an online data collection tool. The expert panel rated the equivalence of the translation to the original English text as either yes or no. The expert panel included nurses, physicians and researchers, with post-graduate training, who had at least a decade of experience in either geriatric research or clinical practice. We assess the relevance of the VPCC measure to geriatric care in Vietnam by using the ratings from the bilingual expert panel to calculate content validity index (CVI) scores at both the item (I-CVI) and scale (S-CVI) levels. [28] The CVI process has been documented to predict potentially problematic survey items. [29] While the S-CVI measures the proportion of the survey judged relevant, the I-CVI measures the proportion of agreement on the relevance of each item. S-CVI was calculated as the averages of all the item level CVIs (S-CVI/Ave).^28^ The modified kappa (*K*m) statistic, which accounts for the probability of some chance agreement among experts, was derived from the I-CVI score. [30] An instrument with *K*m statistic above 0.74, I-CVI score of at least 0.78, or S-CVI/Ave score of at least 0.90 has excellent content validity. [31].

Squire and colleagues, [32] adapted the CVI process to measure translation equivalence at the item (TI-CVI) and scale (TS-CVI/Ave) levels. We used the expert panel ratings to calculate TI-CVI and TS-CVI/Ave scores to evaluate the translation equivalence of the VPCC measure. We used Microsoft Excel 2016 for the CVI calculations.

### Piloting the patient-centered care measure

The study was approved by the Institutional Review Boards at Johns Hopkins Bloomberg School of Public Health and Hanoi University of Public Health. Approvals were obtained from the administrative leaders of each health facility prior to data collection. Interviewers participated in a two-day training, including pretest at a health facility. The finalized VPCC measure incorporated feedback from the expert panel and pretest.

Interviewers administered the VPCC measure to healthcare providers between March and April 2019. In addition to the VPPC measure, the survey included sections on geriatric knowledge assessment using the Vietnamese version of the Knowledge about Older Patients-Quiz (VKOP-Q) [11] and demographic characteristics of the respondents. The Knowledge about Older Patients-Quiz is an instrument to assess gaps in the geriatric knowledge among healthcare providers. It contains 30 dichotomous (true or false) statements to measure the knowledge of healthcare providers about the appropriate care for hospitalized older adults, as well as the healthcare providers’ certainty in their responses. [33] The study sample size was calculated with statistical power analysis using the effect size, probability of not having a type II error (power), and the probability of committing a type I error (alpha). [34] Effect size is the difference in means among comparison groups of healthcare providers. Power and alpha were set at 0.80 and 0.05, respectively. A minimum sample size of 79 was required to avoid type II error with a medium effect size.

We used convenience sampling strategy to select the health facilities from two urban districts and three suburban districts, across the three levels (commune, district/provincial, and central) of healthcare facilities in Vietnam. Communes provide basic health services, while patients who require specialized care are referred to district/provincial or central health facilities. The average number of eligible respondents per health facility at the commune, district/provincial, and central levels were 5, 76, and 135 healthcare providers, respectively. [35, 36] Quota sampling method was used so that the sample was proportional to the health facility size. Commune, district/provincial, and central levels were assigned maximum values of 2, 10, and 20 participants per health facility, respectively.

### Data Analysis

Data were entered into a form on Kobo Toolbox, a secure web-based application for data collection and management. [37] Data entry was verified by two researchers. Data were exported to Stata 15 software for analysis. Data were coded based on the instructions provided by Sidani et al. [38] The items were grouped into 3 subscales: holistic care (9 items), collaborative care (7 items), and responsive care (4 items).

Summed indexes were calculated for each subscale. Average index scores were computed by dividing the summed indexes by the total number of questions for each subscale. Possible values for the averaged index scores ranged from 0 to 5. Higher scores indicated a more favorable assessment of the implementation of patient-centered care. Only one missing data was observed and it was handled by listwise deletion for that subscale.

Measures of central tendency and dispersion were computed for each of the index scores. The Shapiro-Wilk test was used to evaluate deviance from a normal distribution for the averaged index scores. The observed index scores were left-skewed and did not pass the normality tests. Hence, we used nonparametric tests, which are appropriate when there is a violation of parametric assumptions. [39] Mann-Whitney *U* and Kruskal-Wallis tests were used to assess intergroup differences for each item and the average index scores. We tested the null hypothesis that comparison groups were based on the work experiences and demographics of healthcare providers. We examined differences in means by occupation, post-graduate education status, health facility level, and prior geriatric training. We described the PCC themes of the higher and lower rated activities among the VPCC measure.

Examination of the distributions revealed the majority of the average index scores ranged from 3 to 5, further supporting the need for binary rather than continuous dependent variable analyses. Therefore, the average index scores were collapsed to create binary variables. The collapsed index scores were coded as ≥ 0 and < 4 = 0 for low implementation and ≥ 4 and ≤ 5 = 1 for high implementation. The binary index variables were used in the multiple logistic regression analyses.

Multiple logistic regression models were specified to test the a priori null hypothesis that geriatric knowledge is not different among healthcare providers with perception of high implementation compared with low implementation of PCC measures. Odds ratios (OR) were used to measure the association between the geriatric knowledge score and the dependent variables, adjusting for the characteristics of healthcare providers. The variables in the regression models were defined in Table 1. A *p*-value equal to or lower than 0.05 was regarded as statistically significant.

**Table 1:** List of variables and definitions

Variables	Definitions
Holistic care subscale	Dependent variable: ≥0 and < 4 = 0 for low implementation; ≥4 and ≤ 5 = 1 for high implementation
Collaborative care subscale	Dependent variable: ≥0 and < 4 = 0 for low implementation; ≥4 and ≤ 5 = 1 for high implementation
Responsive care subscale	Dependent variable: ≥0 and < 4 = 0 for low implementation; ≥4 and ≤ 5 = 1 for high implementation
Patient centered care scale	Dependent variable: ≥0 and < 4 = 0 for low implementation; ≥4 and ≤ 5 = 1 for high implementation
Knowledge score	Continuous from 0–30 using Vietnamese Knowledge about Older Patients Quiz (VKOP-Q)
Occupation	Nurses (ref); Doctor
Sex	Males(ref); Females
Any post-graduate training	No (ref); Yes
Years of experience	0–4 years (ref); 5 + years
Number of aging patients per day	Continuous

## Results

### Content and translation validation

**Fig. 1:**
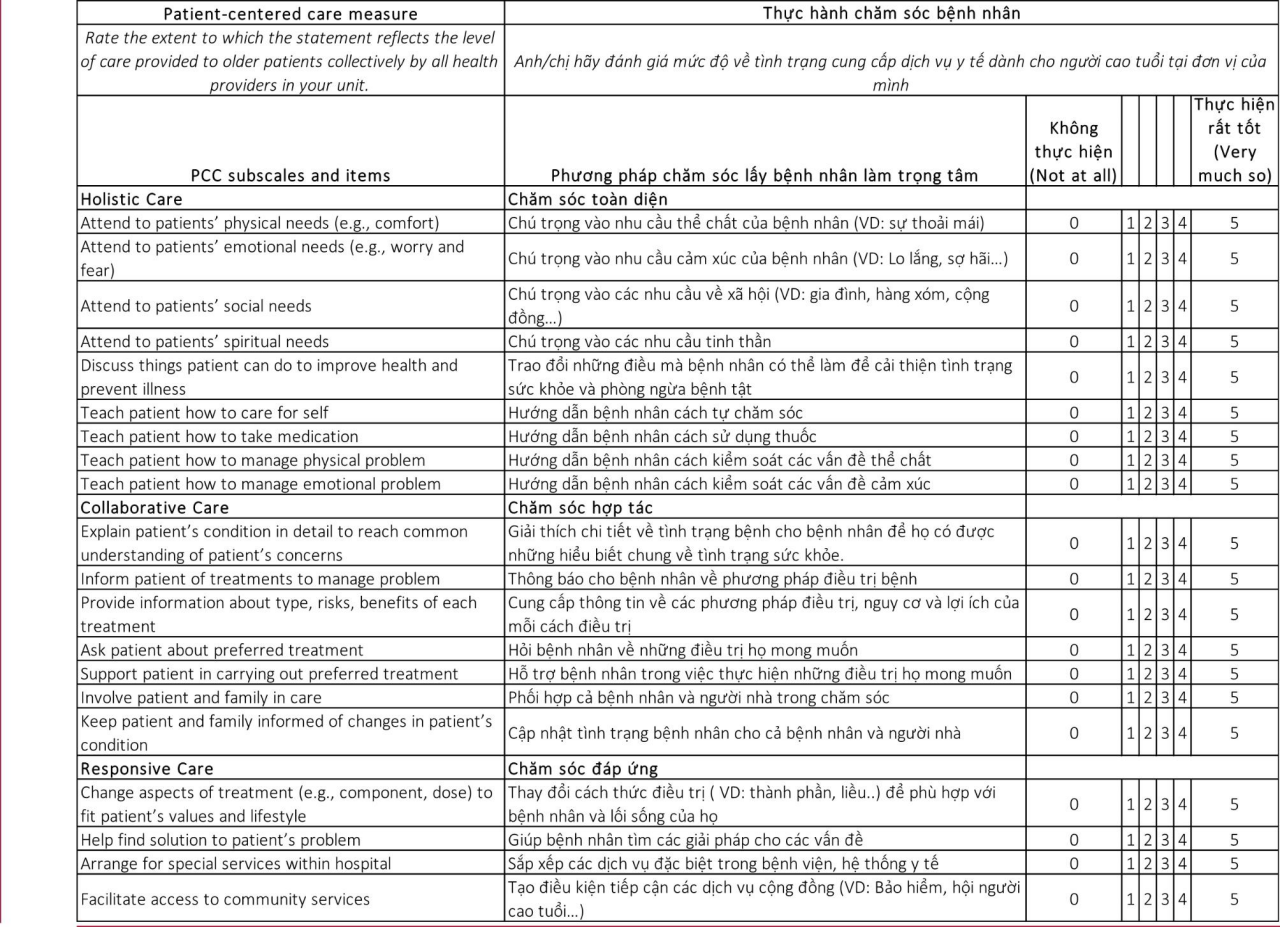
The Vietnamese Patient Centered Care (VPCC) Measure

Figure 1 shows the final VPCC measure.

Table 2 presents the scale-level results of the CVI indices. *K*m values were identical to the CVI indices. On the item level, all 20 questions had excellent validity ratings. None of the I-CVI was below 0.50, which is the criteria for rejection. The S-CVI/Ave was 0.96, which translates to excellent overall content validity. Similarly, all of the items had TI-CVI scores ≥ 0.78, which means the translation equivalence was rated excellent. The TS- CVI/Ave was 0.94, which means that the overall translation equivalence of the instrument was rated excellent.

**Table 2:** Content and Translation Validity of the PCC Measure

N	Content of the item	Content Relevance				Translation Equivalence			
		Expert (N)	Rating ≥ 3 (N)	I-CVI	Evaluationi	Expert (N)	Rating ≥ 3 (N)	TI-CVI	Evaluation^i^
1	Attend to patients’ physical needs (e.g., comfort)	7	7	1.00	Excellent	7	7	1.00	Excellent
2	Attend to patients’ emotional needs (e.g., worry and fear)	7	7	1.00	Excellent	7	7	1.00	Excellent
3	Attend to patients’ social needs	7	7	1.00	Excellent	7	7	1.00	Excellent
4	Attend to patients’ spiritual needs	7	7	1.00	Excellent	7	6	0.86	Excellent
5	Discuss things patient can do to improve health and prevent illness	7	7	1.00	Excellent	7	6	0.86	Excellent
6	Teach patient how to care for self	7	6	0.86	Excellent	7	7	1.00	Excellent
7	Teach patient how to take medication	7	7	1.00	Excellent	7	7	1.00	Excellent
8	Teach patient how to manage physical problem	7	6	0.86	Excellent	7	6	0.86	Excellent
9	Teach patient how to manage emotional problem	7	7	1.00	Excellent	7	7	1.00	Excellent
10	Explain patient’s condition in detail to reach common understanding of patient’s concerns	7	7	1.00	Excellent	7	7	1.00	Excellent
11	Inform patient of treatments to manage problem	7	7	1.00	Excellent	7	7	1.00	Excellent
12	Provide information about type, risks, benefits of each treatment	7	7	1.00	Excellent	7	6	0.86	Excellent
13	Ask patient about preferred treatment	7	6	0.86	Excellent	7	6	0.86	Excellent
14	Support patient in carrying out preferred treatment	7	6	0.86	Excellent	7	6	0.86	Excellent
15	Involve patient and family in care	7	6	0.86	Excellent	7	6	0.86	Excellent
16	Keep patient and family informed of changes in patient’s condition	7	7	1.00	Excellent	7	7	1.00	Excellent
17	Change aspects of treatment (e.g., component, dose) to fit patient’s values and lifestyle	7	7	1.00	Excellent	7	6	0.86	Excellent
18	Help find solution to patient’s problem	7	7	1.00	Excellent	7	6	0.86	Excellent
19	Arrange for special services within hospital	7	7	1.00	Excellent	7	7	1.00	Excellent
20	Facilitate access to community services	7	6	0.86	Excellent	7	7	1.00	Excellent
		S-CVI/Ave		0.96		TS-CVI/Ave		0.94	

^i^Evaluation criteria for the level of validity: excellent validity = I-CVI ≥ 0.78 [40].

### Patient-centered geriatric care among healthcare providers

The VPCC was administered to 112 nurses and physicians in 30 facilities across Hanoi. The demographics of the participants in the pilot were summarized in Table 3.

**Table 3:** Descriptive statistics of pilot study population

Variables	Total (N = 112)	
	**N**	%
Occupation		
Nurses	71	63.4
Physicians	41	37.6
Females	83	74.1
Any post-graduate training	31	27.7
Years of experience, *mean (SD)*	112	12.6 (0.8)
Number of aging patients per day, *mean (SD)*	110	10 (0.9)
Urban location	53	47.3
Districts		
Cau Giay	18	16.1
Dong Anh	35	31.3
Dong Da	11	9.8
Dan Phuong	40	35.7
Long Bien	8	7.1
Facility levels		
Commune	43	38.4
District or Provincial	29	25.9
Central	40	35.7
Departments		
Geriatric	19	17
Primary	48	42.9
Internal	26	23.2
Cardiology	16	14.3

The measures of central tendency indicated that healthcare providers perceived they provided a moderately high level of patient-centered geriatric care. The means for the complete scale and subscales were presented in Table 4.

**Table 4:** Mean and 95% Confidence Intervals of PCC Practices

Patient-Centered Care Measures	Mean	95% CI
Holistic care	3.91	3.80–4.03
Collaborative care	4.11	3.99–4.22
Responsive care	3.71	3.57–3.86
Patient-Centered Care	3.94	3.83–4.04

**Fig. 2:**
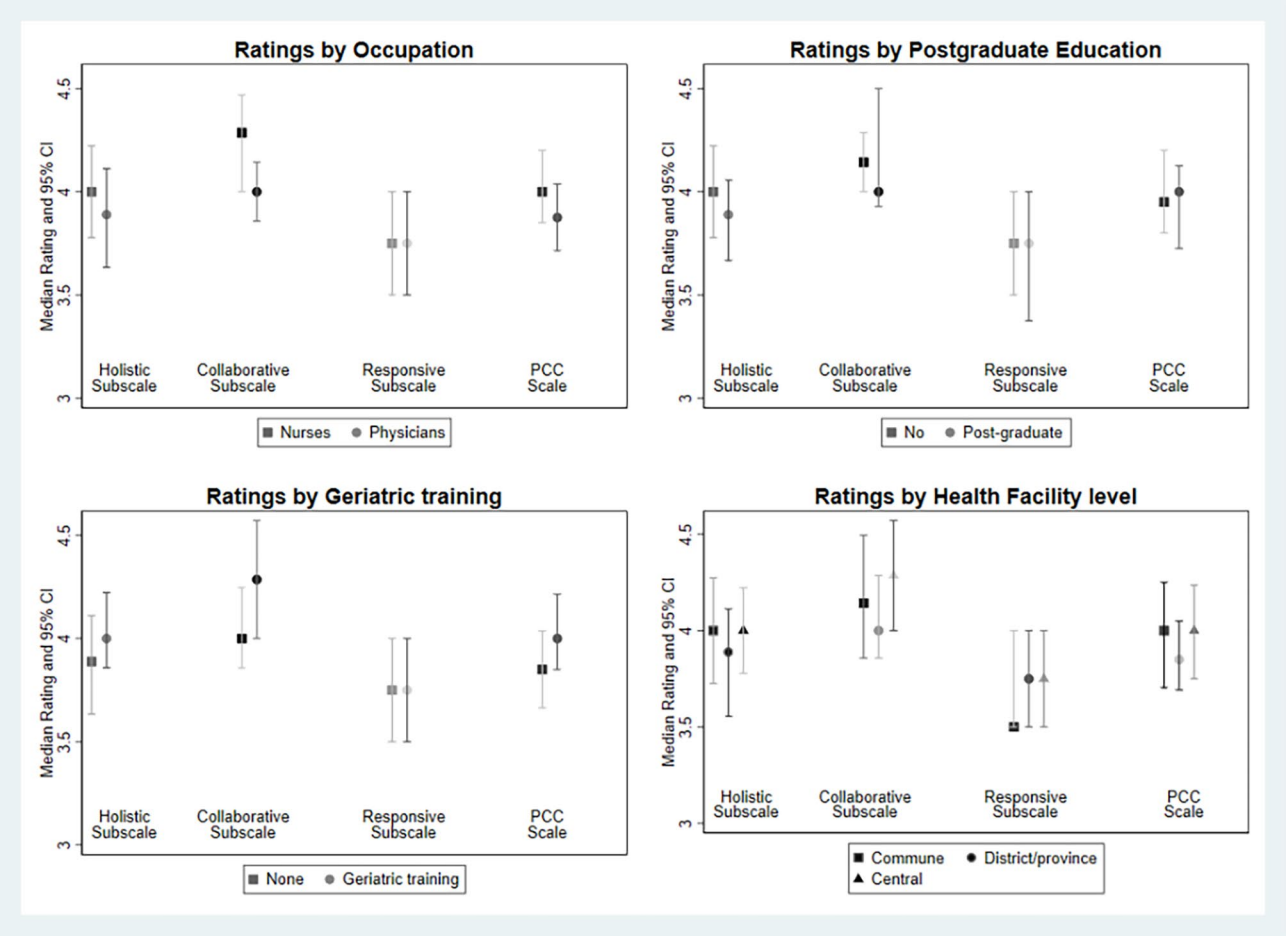
Comparison of Median Scores for PCC Measures by Groups

The highest rated subscale was for collaborative care, while the lowest rated subscale was for responsive care. The differences between the highest and lowest-rated subscales were confirmed with the statistically significant Friedman test and Wilcoxon signed rank sum test. Results of the comparison of median scores for the PCC and its subscales were presented in Fig. 2. The self-assessment of patient-centered care was largely homogeneous across groups and the Kruskal-Wallis tests were not significant.

The five higher and lower rated activities were listed in Table 5. Similar to the subscale findings, the highest-rated items were activities grouped in either the provision of information or collaborative care subscales. The lowest-rated items were spread across the subscales, except for collaborative care. The lowest rated activities were related to the provision of psychosocial care and coordination within and beyond the health system.

**Table 5:** Perception of patient-centered care among healthcare provider

Subscale	PCC activities	Mean	95% CI
**Higher scored PCC activities **			
Holistic care: Provision of information	Teach patient how to take medication	4.4	4.2—4.5
Collaborative care	Explain patient’s condition in detail to reach common understanding of patient’s concerns	4.3	4.2—4.5
Collaborative care	Inform patient of treatments to manage problem	4.3	4.2—4.5
Holistic care: Provision of information	Teach patient how to care for self	4.3	4.2—4.4
Holistic care: Provision of information	Discuss things patient can do to improve health and prevent illness	4.2	4.1—4.4
**Lower scored PCC activities**			
Responsive care	Arrange for special services within hospital or health system	3.2	2.9—3.5
Holistic care: Attendance to patients’ needs	Attend to patients’ social needs (e.g., family, neighbors, community, etc.)	3.4	3.2—3.6
Holistic care: Provision of information	Teach patient how to manage emotional problem	3.6	3.4—3.7
Responsive care	Help find solution to patient’s problem	3.7	3.5—3.9
Holistic care: Attendance to patients’ needs	Attend to patients’ spiritual needs	3.8	3.6—4.0
Responsive care	Facilitate access to community services	3.8	3.6—4.0

Results from the multivariate logistic regression models for the four binary index scores were presented in Table 6. After controlling for healthcare provider characteristics, the odds of the perception of high implementation of collaborative care were increased by 21% for each increase in geriatric knowledge score. We fail to reject the null hypotheses for holistic care, responsive care and PCC.

**Table 6:** Logistic Regression Models for the PCC scale and subscales^i^

Variables	Holistic care		Collaborative care		Responsive care		Patient-centered care	
	Adjusted Odds Ratio	95% CI	Adjusted Odds Ratio	95% CI	Adjusted Odds Ratio	95% CI	Adjusted Odds Ratio	95% CI
**Geriatric knowledge score (VKOP-Q)**	1.01	0.86–1.19	1.21**	1.02–1.44	1.00	0.83–1.20	1.01	0.86–1.20
**Number of older patients/day**	1.01	0.97–1.06	0.98	0.94–1.03	1.02	0.97–1.07	0.99	0.94–1.04
**Years of experience**								
5 + years	1.22	0.45–3.33	1.47	0.51–4.19	1.92	0.58–6.32	1.27	0.45–3.57
**Any post-graduate education**								
Yes	0.55	0.21–1.44	0.71	0.27–1.88	0.41	0.13–1.25	1.02	0.39–2.64
**Occupation**								
Doctor	0.75	0.30–1.89	0.34**	0.13–0.88	0.91	0.32–2.56	0.63	0.24–1.61
**Sex**								
Female	1.63	0.64–4.16	0.69	0.26–1.78	2.13	0.77–5.91	1.82	0.70–4.71

^i^ all models were adjusted for interviewer effect | **p-value < 0.01.

## Discussion

This study is the first to validate a culturally relevant instrument to measure the practice of patient-centered geriatric care in Vietnam. The PCC measure was selected for translation because it specifies activities that operationalize the process of providing comprehensive patient-centered care, instead of the general perspectives of healthcare providers or single domains of patient-centered care. [21, 41, 42] Process measures are sensitive to the differences in the quality of care and have the advantage of reproducibility. [43].

The Vietnamese translation of the PCC measure followed international standards for cross-cultural adaptation of surveys in health services research to reduce the threats to data validity and improve instrument reliability. [27, 32, 44, 45] The S-CVI/Ave and TS-CVI/Ave scores for the VPCC measure demonstrated excellent content validity and translation equivalence. As Vietnam’s population continues to age rapidly, the validated VPCC measure may be used to assess the process and implementation of patient-centered care from the perspectives of healthcare providers, patients and their families. [23].

Healthcare providers were the focus of the pilot study because they are active agents in the delivery of quality care. [46, 47] Overall, healthcare providers had a moderately high assessment of their implementation of patient-centered care. The high ratings for PCC was congruent with studies among similar professional groups in Canada. [24, 38] However, the high PCC scores may reflect the documented tendencies of healthcare professionals to overrate their performance or provide the expected answer on self-report instruments. [48, 49]

The highest rated subscales were the practice of collaborative care and the provision of information. We previously reported that these healthcare providers scored highly on VKOP-Q items related to the knowledge of appropriate family interventions for geriatric care. [11] Higher geriatric knowledge score was associated with increasing odds of high implementation of collaborative care, which suggests some knowledge translation among the healthcare providers in this study. Provision of health information and shared-decision making, which is an outcome of collaborative care, are particularly important to promote treatment adherence and improved health outcomes among aging patients with multimorbidity. [50, 51] Healthcare providers’ perceived excellence and confidence in providing information to patients is necessary for improving health literacy, trust in provider-patient relationships, enabling aging patients to participate in shared decision-making with the healthcare provider, and overall satisfaction with care. [52–54].

The lowest rated subscales in the pilot study were the implementation of holistic attendance to patients’ needs and responsive care. Both subscales reflect the individualization of treatment to meet the patient’s needs, resources, and preferences, during and after discharge from the hospital. Specifically, the lowest rated activities within these subscales pertained to meeting the social and emotional needs of patients, which were traditionally beyond the health system. The provision of holistic attendance to patients’ needs and responsive care are crucial for maintaining the dignity of aging patients and ensuring their continuity of care. [55] These findings on perceived low practice of holistic and responsive care in the pilot study were corroborated by analysis of Vietnam’s national aging survey which showed that aging adults with multimorbidity had higher odds of unmet health needs, even among those who received medical care in the past year. [11] In the same analysis, it was reported that healthcare did not reduce the risk of unmet needs for assistance among aging adults, which suggests fragmented coordination between social and healthcare systems. These perceived gaps in the implementation of holistic care were documented in another study, which reported that the healthcare providers prioritized physical needs, but patients wanted to discuss their feelings and how to manage psychosocial concerns. [23].

Furthermore, an aspect of holistic care is addressing psychosocial needs, such as teaching patients how to manage emotional and social problems of anxiety or social isolation. Patients with met psychosocial needs are more likely to feel prepared for discharge and recovery outside the hospital. [56, 57] Emotional and social needs encompass elder mistreatment, which is under-recognized and associated with somatic symptoms, such as pain. [58, 59] The provision of holistic care may increase the probability of connecting vulnerable aging patients with appropriate information and care. [2] Without appropriate training, healthcare providers may not feel confident about negotiating the balance between patient-centered care and cultural competency, especially related to psychosocial needs that are considered culturally sensitive. [60].

The lower-rated activities for responsive care were related to service coordination for aging people across different levels within and beyond the health sector. Poor service coordination is interconnected with the low scores on addressing the social and emotional needs of aging patients because healthcare providers need to lean on existing networks of multisector services to facilitate timely and appropriate referrals to holistically meet the needs of aging patients. [42, 61]

## Limitations and Future Research

The convenience sampling of healthcare providers from Hanoi, which is mostly urban and suburban, is susceptible to selection bias. In addition, healthcare providers were not recruited from the private sector and health facilities managed by other ministries, including the military health system. Consequently, the result may not be generalizable to other healthcare providers. The inclusion of at least two health facilities for each facility level is likely to have broadened healthcare provider selection and potentially avoided some of the selection bias. Relying on the clinical supervisors to facilitate recruitment may have compounded the bias and threatened the internal validity of the study. However, the demographics, clinical roles and experience level of the respondents were varied.

Self-rated assessment of healthcare providers may not be congruent with external observations of their performance. [62, 63] Studies have documented both discordances and congruencies in the perceptions of patient-centered care by healthcare providers and patients, which highlights the need for future studies on the perception of patient-centered care among aging people in Vietnam. [64, 65] Furthermore, hypothesis test results for knowledge translation in this pilot sample depended on the assumption that PCC implementation was best measured as a binary variable. In a larger sample, the hypothesis test results could be robust to other ways of coding the average index scores.

We did not collect information on the established processes for coordinated care within and across sectors at the participating health facilities, and lack of such information could undermine the ability of healthcare providers to excel in this aspect of patient-centered care. Studies have documented teamwork and established care pathways as critical ingredients to efficient care coordination for older patients. [66, 67]

The use of interviewers may have increased the risk of social desirability bias in the ratings. However, the confidentiality of responses was communicated to respondents and the multivariate logistic regression models adjusted for interviewer effect.

Future research should include additional investigation of the psychometric properties of the VPCC measure, as well as its relevance to different population groups and healthcare contexts in Vietnam.

## Conclusion

This study successfully adapted and validated the cross-cultural relevance of the PCC measure for geriatric care in Vietnam. In our pilot study, the highest-rated subscales were the provision of information and collaborative care, while the lowest-rated subscales were the holistic attendance to patients’ needs and responsive care. Attention to the psychosocial needs of aging patients and poor coordination of care within and beyond the health system were the lowest-rated PCC activities by healthcare providers in this pilot study. Despite the limitations of this study, it revealed the need for further assessment of the practice of patient-centered geriatric care across health facilities in Vietnam.

## Data Availability

The datasets used during the current study are available from the corresponding author on reasonable request.

## Abbreviations

CVI: Content Validity Indexing
KOP-Q: Knowledge about Older Patient’s Quiz
MLR: Multiple Logistic Regression
PCC: Patient-Centered Care
VKOP-Q: Vietnamese Knowledge about Older Patient’s Quiz
VPCC: Vietnamese Patient-Centered Care

## References

[1] World Health Organization. Decade of healthy ageing: Baseline report. 2020.

[2] World Health Organization. World report on ageing and health. World Health Organization; 2015.

[3] Vasto S, Scapagnini G, Bulati M (2010). Biomarkers of aging. Front Biosci.

[4] Steves CJ, Spector TD, Jackson SH (2012). Ageing, genes, environment and epigenetics: what twin studies tell us now, and in the future. Age Ageing.

[5] Dai W, Lu S. The “Socialization of elderly hospitalization” in China: Development, problems, and solutions. Journal of Social Service Research. 2018;44(4):518–528. 10.1080/01488376.2018.1477696. Accessed Sep 23, 2019.

[6] Dai W. Is China facing the social risks associated with reliance on hospitalization for the care of the elderly with chronic diseases? The International Journal of Health Planning and Management. 2019;34(2):794–805. https://onlinelibrary.wiley.com/doi/abs/10.1002/hpm.2738. Accessed Sep 23, 2019. 10.1002/hpm.2738. 10.1002/hpm.2738 30680806

[7] Tricco AC, Antony J, Ivers NM (2014). Effectiveness of quality improvement strategies for coordination of care to reduce use of health care services: a systematic review and meta-analysis. CMAJ.

[8] Shi L, Makinen M, Lee D (2015). Integrated care delivery and health care seeking by chronically-ill patients–a case-control study of rural henan province, China. Int J Equity Health.

[9] United Nations. World population ageing 2019 (ST/ESA/SER. A/444). 2020.

[10] Glinskaya EE, De Feige K, Vu Thi AI. LH, Vietnam: Adapting to an aging society. World Bank Group. 2021.

[11] Adetunji O. Assessing the need for integrated and person-centered care for the elderly in vietnam. Johns Hopkins University; 2020.

[12] Vietnam Women’s Union. Vietnam national aging survey (VNAS) 2011: Key findings. Project VIE022, Vietnam Women’s Union. 2012.

[13] Socialist Republic of Vietnam, Ministry of Health. Decision 2151/QD-BYT. https://english.luatvietnam.vn/decision-no-2151-qd-byt-dated-june-04-2015-of-the-ministry-of-health-approving-the-plan-for-implementation-of-innovation-of-serving-manner-and-atti-95515-doc1.html. Updated 2015.

[14] Vietnam News Agency. Revised law on medical examination and treatment promulgated. https://en.vietnamplus.vn/revised-law-on-medical-examination-and-treatment-promulgated/247806.vnp. Updated 2023. Accessed Feb 4, 2023.

[15] Evans JM, Kiran PR, Bhattacharyya OK (2011). Activating the knowledge-to-action cycle for geriatric care in India. Health Res policy Syst.

[16] Avedis Donabedian (2005). Evaluating the quality of medical care. Milbank Q.

[17] Johri M, Beland F, Bergman H (2003). International experiments in integrated care for the elderly: a synthesis of the evidence. Int J Geriatr Psychiatry.

[18] World Health Organization. WHO global strategy on people-centred and integrated health services: Interim report. 2015.

[19] World Health Organization. Quality of care: A process for making strategic choices in health systems. 2016.

[20] Sidani S, Fox M (2014). Patient-centered care: clarification of its specific elements to facilitate interprofessional care. J Interprof Care.

[21] Sidani S, Collins L, Harbman P (2014). Development of a measure to assess healthcare providers’ implementation of patient-centered care. Worldviews on Evidence‐Based Nursing.

[22] Agency for Healthcare Research and Quality. Module 6. assessing practice systems. https://archive.ahrq.gov/ncepcr/tools/pf-handbook/mod6.html. Updated 2013. Accessed July 15, 2022.

[23] Sidani S, Soeren Mv, Hurlock-Chorostecki C, Reeves S, Fox M, Collins L. Health professionals’ and patients’ perceptions of patient-centered care: A comparison. European Journal for Person Centered Healthcare. 2016;4(4):641–649. http://ubplj.org/index.php/ejpch/article/view/1177. Accessed Oct 28, 2018. 10.5750/ejpch.v4i4.1177.

[24] Dahlke S, Hunter KF, Negrin K, Reshef Kalogirou M, Fox M, Wagg A. The educational needs of nursing staff when working with hospitalised older people. J Clin Nurs. 2018;0(0). 10.1111/jocn.14631. https://www.ncbi.nlm.nih.gov/pubmed/30039614. 10.1111/jocn.14631 30039614

[25] Streiner DL, Norman GR, Cairney J (2015). Health measurement scales: a practical guide to their development and use.

[26] World Health Organization. WHO process of translation and adaptation of research instruments. Updated 2010. Accessed Dec 18, 2018.

[27] Maneesriwongul W, Dixon JK (2004). Instrument translation process: a methods review. J Adv Nurs.

[28] Polit DF, Beck CT (2006). The content validity index: are you sure you know what’s being reported? Critique and recommendations. Res Nurs Health.

[29] Olson K (2010). An examination of questionnaire evaluation by expert reviewers. Field Methods.

[30] Fleiss JL, Levin B, Paik MC (1981). The measurement of interrater agreement. Stat methods rates proportions.

[31] Polit DF, Beck CT, Owen SV (2007). Is the CVI an acceptable indicator of content validity? Appraisal and recommendations. Res Nurs Health.

[32] Squires A, Aiken LH, van den Heede K (2013). A systematic survey instrument translation process for multi-country, comparative health workforce studies. Int J Nurs Stud.

[33] Dikken J, Hoogerduijn JG, Kruitwagen C, Schuurmans MJ. Content validity and psychometric characteristics of the “Knowledge about older patients quiz” for nurses using item response theory. Journal of the American Geriatrics Society. 2016;64(11):2378–2383. https://onlinelibrary.wiley.com/doi/abs/10.1111/jgs.14476. Accessed Oct 28, 2018. 10.1111/jgs.14476. 10.1111/jgs.14476 27627575

[34] Cohen J. Statistical power analysis for the behavioral sciences. Routledge; 2013.

[35] The World Bank. Quality and equity in basic health care services in vietnam: Findings from the 2015 vietnam district and commune health facility survey. The World Bank. 2016.

[36] World Health Organization. Human resources for health country profiles: Viet nam. 2016.

[37] KoBo Inc. KOBO toolbox. https://www.kobotoolbox.org/kobo/2019.

[38] Sidani S, Reeves S, Hurlock-Chorostecki C, van Soeren M, Fox M, Collins L (2018). Exploring differences in patient-centered practices among healthcare professionals in acute care settings. Health Commun.

[39] Lehmann EL, D’Abrera H, Nonparametrics (1998). Statistical methods based on ranks (rev. ed.) Prentice-hall. Englewood Cliffs NJ.

[40] Halek M, Holle D, Bartholomeyczik S (2017). Development and evaluation of the content validity, practicability and feasibility of the innovative dementia-oriented assessment system for challenging behaviour in residents with dementia. BMC Health Serv Res.

[41] Gachoud D, Albert M, Kuper A, Stroud L, Reeves S (2012). Meanings and perceptions of patient-centeredness in social work, nursing and medicine: a comparative study. J Interprof Care.

[42] Andrew M, Briggs (2018). Islene Araujo de Carvalho. Actions required to implement integrated care for older people in the community using the world health organization’s ICOPE approach: a global delphi consensus study. PLoS ONE.

[43] Mant J (2001). Process versus outcome indicators in the assessment of quality of health care. Int J Qual Health Care.

[44] Beaton DE, Bombardier C, Guillemin F, Ferraz MB (2000). Guidelines for the process of cross-cultural adaptation of self-report measures. Spine.

[45] Gjersing L, Caplehorn JR, Clausen T (2010). Cross-cultural adaptation of research instruments: Language, setting, time and statistical considerations. BMC Med Res Methodol.

[46] Akachi Y, Kruk ME (2017). Quality of care: measuring a neglected driver of improved health. Bull World Health Organ.

[47] Hanefeld J, Powell-Jackson T, Balabanova D (2017). Understanding and measuring quality of care: dealing with complexity. Bull World Health Organ.

[48] MILLER FRANCOL, Franco C, Kumwenda N, Nkhoma W (2002). Methods for assessing quality of provider performance in developing countries. Int J Qual Health Care.

[49] Walfish S, McAlister B, O’Donnell P, Lambert MJ (2012). An investigation of self-assessment bias in mental health providers. Psychol Rep.

[50] Son Y, Yoon H. A concept analysis on patient-centered care in hospitalized older adults with multimorbidity. Journal of Korean Critical Care Nursing. 2019;12(2).

[51] Dhar L, Earnest J, Ali M (2017). A systematic review of factors influencing medication adherence to hypertension treatment in developing countries. Open J Epidemiol.

[52] Shahin W, Kennedy GA, Stupans I (2019). The impact of personal and cultural beliefs on medication adherence of patients with chronic illnesses: a systematic review. Patient Prefer Adherence.

[53] Tobiano G, Marshall A, Bucknall T, Chaboyer W (2015). Patient participation in nursing care on medical wards: an integrative review. Int J Nurs Stud.

[54] Weingart SN, Zhu J, Chiappetta L (2011). Hospitalized patients’ participation and its impact on quality of care and patient safety. Int J Qual Health Care.

[55] World Health Organization. Continuity and coordination of care: A practice brief to support implementation of the WHO framework on integrated people-centred health services. 2018.

[56] Fitzgerald Miller J, Piacentine LB, Weiss M (2008). Coping difficulties after hospitalization. Clin Nurs Res.

[57] Hesselink G, Flink M, Olsson M (2012). Are patients discharged with care? A qualitative study of perceptions and experiences of patients, family members and care providers. BMJ Qual Saf.

[58] Jorge Fernandes Soares J, Viitasara E, Macassa G (2014). The impact of psychological abuse on somatic symptoms: a study of older persons aged 60–84 years. J Adult Prot.

[59] Park H. Living with ‘hwa-byung’: The psycho-social impact of elder mistreatment on the health and well-being of older people. Aging Ment Health. 2014;18(1):125–128. Accessed May 16, 2018. 10.1080/13607863.2013.814103. 10.1080/13607863.2013.814103 23815590

[60] Nielsen LS, Angus JE, Howell D, Husain A, Gastaldo D (2015). Patient-centered care or cultural competence: negotiating palliative care at home for chinese canadian immigrants. Am J Hospice Palliat Medicine®.

[61] Araujo de Carvalho I, Epping-Jordan J, Pot AM (2017). Organizing integrated health-care services to meet older people’s needs. Bull World Health Organ.

[62] Mullan BA, Kothe EJ (2010). Evaluating a nursing communication skills training course: the relationships between self-rated ability, satisfaction, and actual performance. Nurse Educ Pract.

[63] Pollard S, Bansback N, Bryan S (2015). Physician attitudes toward shared decision making: a systematic review. Patient Educ Couns.

[64] Poirier P, Sossong A (2010). Oncology patients’ and nurses’ perceptions of caring. Can Oncol Nurs Journal/Revue canadienne de soins infirmiers en oncologie.

[65] Sossong A, Poirier P (2013). Patient and nurse perceptions of caring in rural united states. Int J Hum Caring.

[66] Bodenheimer T. Coordinating care — A perilous journey through the health care system. New England Journal of Medicine. 2008;358(10):1064–1071. 10.1056/NEJMhpr0706165. Accessed Sep 23, 2019. 10.1056/NEJMhpr0706165 18322289

[67] McDonald KM, Sundaram V, Bravata DM et al. Closing the quality gap: A critical analysis of quality improvement strategies (vol. 7: Care coordination). 2007. 20734531

